# Improved Frequency Sweep Keying CDMA Using Faster R-CNN for Extended Ultrasonic Crosstalk Reduction

**DOI:** 10.3390/s23239550

**Published:** 2023-12-01

**Authors:** Ga-Rin Park, Sang-Ho Park, Kwang-Ryul Baek

**Affiliations:** 1 School of Electric and Electronic Engineering, Pusan National University, Busan 46241, Republic of Korea; parkhm7446@pusan.ac.kr (G.-R.P.); propia@pusan.ac.kr (S.-H.P.); 2 School of Electronic Engineering, Pusan National University, Busan 46241, Republic of Korea

**Keywords:** frequency sweep keying, ultrasonic CDMA, chirp ultrasound, ultrasonic ranging system, ultrasonic crosstalk reduction, autonomous driving, front–rear collision avoidance

## Abstract

Ultrasonic sensors are inexpensive and provide highly accurate measurements, even with simple hardware configurations, facilitating their use in various fields. When multiple ultrasonic sensors exist in the measurement space, crosstalk occurs due to other nodes, which leads to incorrect measurements. Crosstalk includes not only receiving homogeneous signals from other nodes, but also overlapping by other signals and interference by heterogeneous signals. This paper proposes using frequency sweep keying modulation to provide robustness against overlap and a faster region-based convolutional neural network (R-CNN) demodulator to reduce the interference caused by heterogeneous signals. The demodulator works by training Faster R-CNN with the spectrograms of various received signals and classifying the received signals using Faster R-CNN. Experiments implementing an ultrasonic crosstalk environment showed that, compared to on–off keying (OOK), phase-shift keying (PSK), and frequency-shift keying (FSK), the proposed method can implement CDMA even with shorter codes and is robust against overlap. Compared to correlation-based frequency sweep keying, the time-of-flight error was reduced by approximately 75%. While the existing demodulators did not consider heterogeneous signals, the proposed method ignored approximately 99% of the OOK and PSK signals and approximately 79% of the FSK signals. The proposed method performed better than the existing methods and is expected to be used in various applications.

## 1. Introduction

Ultrasonic sensors contain transducers with various frequencies, from 40 kHz transducers commonly used for distance measurement in air to tens of MHz transducers used for non-destructive testing. The measurement resolution ranges from a few centimeters to tens of nanometers, depending on the ultrasonic wavelength. Ultrasonic sensors have been used in various applications, such as distance measurement [1,2,3], positioning [4,5,6], non-destructive testing [7,8], and human physical inspection [9,10,11], because of their diverse measurement resolution. In particular, ultrasonic sensors see widespread use to obtain distance measurements in air because they are less expensive than other sensors and can measure with high accuracy, even with simple hardware configurations. For example, they can be attached to a car and used to implement intelligent safety systems, such as autonomous driving or front and rear collision prevention [12,13,14]. Recently, crosstalk between ultrasonic signals has become a problem due to the increasing number of cars equipped with intelligent safety systems implemented via ultrasound [15,16]. When obtaining measurements in an environment in which multiple ultrasonic sensors exist, ultrasonic signals may not be received, or ultrasonic signals from other sensors may be received due to crosstalk among the sensors. In this case, the measurement error of the ultrasonic sensor may become very large, or the system may fail to operate normally [17].

Several multiple-access methods have been proposed to ensure orthogonality between each signal in an environment with crosstalk among ultrasonic signals. One such method, which is known as time division multiple access (TDMA), ensures orthogonality by having each node measure the time of flight (TOF) at different times [18,19,20]. When using this method, even after the TOF measurement is completed, the ultrasound remains in the medium, so the subsequent TOF measurement may only be conducted once the ultrasound in the medium has disappeared entirely. This significantly increases the time taken to obtain the measurement. The second method, frequency division multiple access (FDMA), ensures orthogonality by having each node perform TOF measurements with different frequencies [21,22]. FDMA may be implemented either by using a wideband ultrasound transducer or by using several narrowband ultrasound transducers. Wideband ultrasound transducers are more difficult to obtain and more expensive than general narrowband ultrasound transducers. When using multiple narrowband ultrasonic transducers, the number of ultrasonic transducers must increase with the number of modes. The third method, code division multiple access (CDMA), ensures orthogonality by assigning different codes to each node [23,24,25,26,27]. It is a commonly used method in ultrasound and is also called coded ultrasound or multi-code ultrasound. CDMA does not increase hardware complexity and has the advantage of reducing the TOF error due to the spread spectrum of pulses during decoding.

In CDMA, modulation/demodulation methods include on–off keying (OOK) [28], phase shift keying (PSK) [29], frequency shift keying (FSK) [30], and frequency sweep keying [31]. Several papers have combined these methods with ultrasound systems to solve the multiple access problem. The modulation method and demodulation algorithm affect the performance of the system because the piezoelectric element of the ultrasonic transducer has resonance characteristics. The authors of [5] proposed an indoor ultrasonic positioning system for multiple robots. The keying method used was OOK. Envelope detection and binarization considering ultrasonic attenuation were performed to improve the demodulator’s performance. The authors of [32] used gold code modulation and proposed using a demodulator with a circular convolution structure. These methods offer robust decoding against environmental noise, but lead to the deterioration of the orthogonality of the code due to demodulation errors occurring when signals from different nodes overlap. The authors of [33] used BPSK to measure indoor distance. A cross-correlation-type demodulator was applied to improve the decoding performance. Although it performed better than ASK in multiple access situations, it increased the number of demodulation errors due to the near–far effect if the difference in the signal strength between nodes was large. In [34], the FSK method was used for indoor localization. The frequency hopping spread spectrum demodulation method was used and incorporated a picoscope, FIR filter, and synchronization process. If the bandwidth of the ultrasonic transducer is sufficient, it has the advantage of small demodulation error and good orthogonality. However, when using a general narrow-band transducer, the transmission and reception signal strength are weak due to the band limiting, making application difficult. The modulation method described in [31], which was previously studied by our research team, sweeps the frequency in the direction of the increase or decrease around the center frequency. Demodulation is performed by correlating the received signal and the reference bit mask in the spectrogram. Frequency sweep keying is robust to signal overlap compared to OOK, PSK, and FSK and can be implemented with a relatively short code length. However, this method has some limitations. First, when a heterogeneous signal is received, it is impossible to distinguish the heterogeneous signal using the existing demodulation method, and a demodulation error can occur. Second, failure to properly set the threshold coefficient may lead to performance degradation. Third, because CDMA is performed on the spectrogram, the time domain resolution is degraded, resulting in low TOF precision.

Ultrasonic signal crosstalk can be caused not only by the homogeneous signals transmitted, but also by heterogeneous signals from unidentified nodes. The previous studies were designed without considering heterogeneous signal input. The same applies to the previous frequency sweep keying method. This paper aimed to ensure that CDMA operates normally by being robust to crosstalk, including overlap, for homogeneous signals and ignoring heterogeneous signals. Therefore, in this paper, we propose a method that uses frequency sweep keying modulation, which is more robust to signal overlap than OOK, PSK, and FSK and uses a faster region-based convolutional-neural-network (R-CNN) [35] demodulation. Frequency sweep keying modulation was proposed in the previous paper and used in this paper, briefly described in relative works, and an improved demodulator is proposed in this study. Therefore, this paper describes a novel demodulator based on Faster R-CNN, and the process of demodulation is described in Section 3. The advantage of the proposed method is that it can ignore heterogeneous signals by classifying the keying type and code. In addition, to solve the problem of low TOF measurement resolution, which is a disadvantage of the demodulation method using a time–frequency spectrogram, the TOF precision is improved by performing signal segmentation in the time domain. In this work, a performance evaluation was conducted by implementing an environment in which ultrasonic crosstalk occurs. Factors such as the SNR and degree of overlap were adjusted, and comparisons were made with related works.

The rest of the paper is organized as follows: Section 2 explains the existing keying methods used in ultrasonic CDMA, including the frequency sweep keying used in this paper. Section 3 describes the demodulation method based on Faster R-CNN and the associated decoding process. Section 4 lists the experimental procedures with ultrasonic sensors and the results of the experiments, while the conclusions and future research directions can be found in Section 5.

## 2. Related Works

### 2.1. Existing Keying Code Division Multiple Access

CDMA is a method that provides orthogonality between signals by assigning different identity (ID) codes to each node to solve the signal interference problem. Even in single-node ultrasound systems, the coded ultrasonic method has been used to improve the SNR of the received signal, improving the measurement distance range and TOF measurement accuracy [36,37]. It has the same advantages in multiple access and is easy to implement in ultrasonic systems. OOK [5,32], PSK [33,38], and FSK [39,40] are used to modulate and demodulate digital codes into analog signals.

OOK is the simplest way to implement CDMA. When the code is ‘1’, the transducer transmits $N_{p}$ ultrasonic pulses; when the code is ‘0’, it does not transmit ultrasound for the same amount of time, $t_{p}=N_{p}/f_{c}$. Here, $N_{p}$ is the number of pulses transmitted per bit, $f_{c}$ is the ultrasonic center frequency, and $t_{p}$ is the transmission time per bit. While this method is easy to implement, the first bit of the ID code must be fixed to code ‘1’ for the TOF measurement. That is, one more bit is always needed in the code length. The demodulator sets a threshold voltage to separate the signal from noise and estimates code ‘1’ when the received signal is greater than the threshold value and code ‘0’ when it is less than the threshold value. Therefore, when the signal overlaps, it is assumed to be code ‘1’, which excludes the overlap of codes ‘0’ and ‘0’. This reduces the orthogonality of the two codes. Hence, long ID codes must be used for normal decoding operation.

PSK is a method of transmitting $N_{p}$ ultrasonic transmission pulses with different phases depending on the ID code. PSK includes binary phase shift keying, which divides a 1 bit code into 180° phase intervals, and quadrature phase shift keying, which divides a 2 bit code into 90° phase intervals. Meanwhile, differential phase shift keying modulates using the phase difference between pulses. PSK is widely used in narrowband ultrasonic transducers because of its good energy efficiency. However, when signals overlap, the phase of the overlapped signal changes depending on the amplitude and phase of the individual signal, which causes demodulation errors to affect the code. Incorrectly estimated codes cannot be predicted, and their correlation with the ID code is low. Hence, decoding is not performed properly, even if the ID code length is long.

FSK is a method that transmits $N_{p}$ ultrasonic transmission pulses at different frequencies based on the center frequency $f_{c}$ according to the ID code. FSK has excellent energy efficiency and modulation and demodulation performance when using wideband ultrasonic transducers. Furthermore, the interference between codes ‘1’ and ‘0’ does not occur when signals overlap. On the other hand, when using a general narrowband ultrasonic transducer, FSK transmits via frequencies that deviate from the center frequency $f_{c}$, resulting in poor energy efficiency, a lower SNR, and a reduced sensing range.

### 2.2. Frequency Sweep Keying Code Division Multiple Access

**Figure 1:**
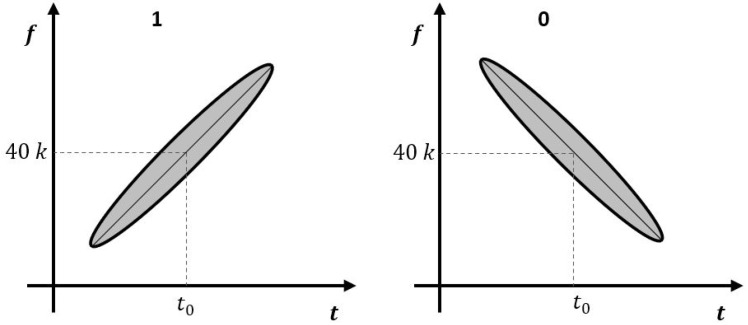
One-bit frequency sweep keying signal on the spectrogram. The x-axis represents time and the y-axis represents frequency. The dotted line represents the center frequency of the ultrasonic sensor and the time at that frequency.

Frequency sweep keying [31] modulates by sweeping the frequency in an increasing or decreasing direction around the center frequency using a general high-Q ultrasonic transducer. Figure 1 shows codes ‘1’ and ‘0’ on a spectrogram modulated using frequency sweep keying. The signal $s_{n,k}\left(t\right)$ corresponding to the n-th bit of the transmission signal is as follows.

(1) $$s_{n,k}\left(t\right)=\sin\left(2\pi f_{k}\left(t\right)\left(t-n∆t\right)\right)\left\{u\left(t-n∆t\right)-u\left(t-\left(n+1\right)∆t\right)\right\}$$

where k is the bit code information with a value of +1 or −1. k is +1 when the bit code is ‘1’ and −1 when the bit code is ‘0’. Δt is the time taken to perform the frequency sweep. $f_{k}\left(t\right)$ represents the sweep frequency according to time and is written as follows:

(2) $$f_{k}\left(t\right)=\frac{∆f}{∆t}k\left(\left(t\;mod\;∆t\right)-\frac{∆t}{2}\right)+f_{c}$$

where $f_{c}$ is the sweep center frequency and is set equal to the center frequency of the ultrasonic transducer. Frequency sweep is performed in the range of the upper limit $f_{c}+\frac{∆f}{2}$ and lower limit $f_{c}-\frac{∆f}{2}$. The transmitted ultrasonic signal $s_{tx}(t)$ using an ideal transducer is expressed as the sum of signals for each bit.

(3) $$s_{tx}\left(t\right)=\sum_{n=1}^{L_{c}}s_{n,k}\left(t\right)$$

The received signal is converted to a spectrogram and demodulated by calculating the cross-correlation using the bit mask and thresholding. Demodulated signals are estimated to have code ‘1’ or code ‘0’, respectively. Each bit mask converts the expected ultrasonic signal corresponding to one bit when the code is ‘1’ or ‘0’ into a time–frequency spectrogram. The threshold value is based on the cross-correlation value between the received signal and the X mask because the result of the correlation operation is proportional to the power of the signal received. The threshold value is given as the product of the cross-correlation value with the X mask and the threshold coefficient set experimentally.

Signals with code ‘0’ or code ‘1’ are estimated through demodulation. Finally, decoding is performed by calculating the correlation between the demodulated signal and the signal that up-sampled the corresponding ID code. This method is more robust to signal overlap than OOK, PSK, and FSK and can be implemented with a relatively short code length.

## 3. Frequency Sweep Keying CDMA Using Faster Region-Based CNN

**Figure 2:**
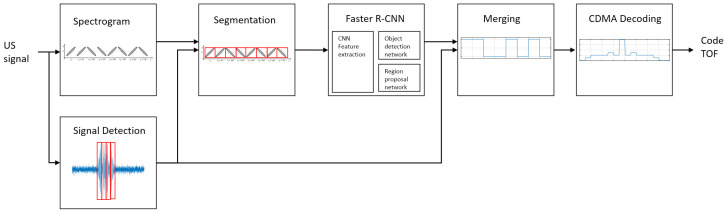
Process of a frequency sweep keying CDMA using Faster R-CNN.

Figure 2 shows the proposed Faster-R-CNN frequency sweep keying demodulation and CDMA decoding process. First, the received signal is converted to a spectrogram. The converted signal is segmented to improve the classification accuracy of Faster R-CNN, and the segmentation area is selected by detecting the signal in the time domain. Segmented signals for each bit are classified according to the code and keying type using Faster R-CNN. Finally, CDMA decoding is performed for the purposes of ID recognition and TOF calculation.

### 3.1. Spectrogram and Segmentation

The proposed demodulator classifies the signal code using Faster R-CNN, merges the results, and outputs the demodulated signal. The segmentation process is implemented to improve the classification performance. After the segmentation range in the time domain has been selected, the signal on the spectrogram is segmented. First, the received ultrasonic signal $s_{rx}[n]$ is converted to a time–frequency spectrogram [41] signal to consider both the time and frequency domains in the classification process. The short-time-Fourier-transform-based spectrogram signal $S_{rx}\left(n,f\right)$ is as follows:

(4) $$S_{rx}\left(n,f\right)={\left|\sum_{m=-\infty }^{\infty }s_{rx}\left[m\right]w\left[m-n\right]e^{-j2\pi fm}\right|}^{2}$$

where $w\left[n\right]$ is the window function, and the following Kaiser window is used.

(5) $$w\left[n\right]=L\cdot w_{0}\left(\frac{L}{N}\left(n-\frac{N}{2}\right)\right)=\frac{I_{0}\left[\beta \sqrt{1-{\left(\frac{2n}{N}-1\right)}^{2}}\right]}{I_{0}\beta },\;0\leq n\leq N$$

where $I_{0}$ is the zeroth-order modified Bessel function, L is the window duration, N is the window length, and β is a parameter that represents the characteristics of the Kaiser window.

Next, the start and end points of the received signal $s_{rx}[n]$ are found for segmentation. Points greater than $6\sigma_{N}$ of noise are considered to be the locations at which the signal exists, and the minimum and maximum values of the index of the signal location are found for the start and end points of the signal. Here, $\sigma_{N}$ is the standard deviation of background noise. The start index $n_{s}$ and end index $n_{e}$ of the signal are as follows:

(6) $$n_{s}={\min(n|s}_{rx}[n]\geq 6\sigma_{N})$$

(7) $$n_{e}={\max(n|s}_{rx}[n]\geq 6\sigma_{N})$$

**Figure 3:**
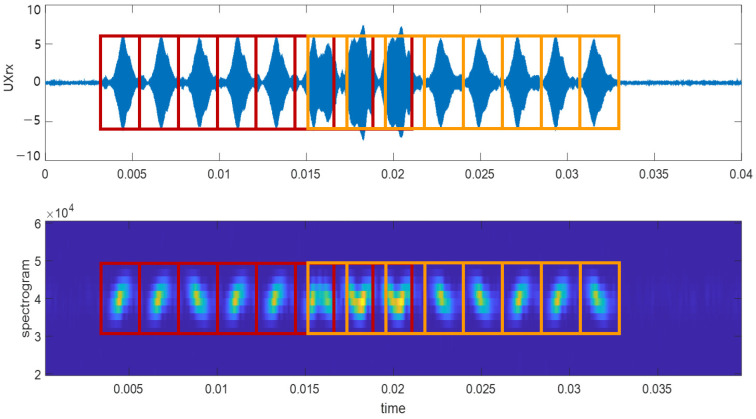
Segmentation results in the time domain (**top**) and spectrogram (**bottom**). Red represents the front signal and orange represents the rear signal.

Two signals, front signal $B_{k}^{f}$ and rear signal $B_{k}^{r}$, are segmented by bit from the start and end points, respectively.

(8) $$B_{k}^{f}=\left\{S_{rx}\left(n,f\right)|n_{s}+\left(k-1\right)w_{c}<n<n_{s}+{kw}_{c}\right\}$$

(9) $$B_{k}^{r}=\left\{S_{rx}\left(n,f\right)|n_{e}-\left(L_{c}-k+1\right)w_{c}<n<n_{e}-\left(L_{c}-k\right)w_{c}\right\}$$

where k is the code bit index and the segmentation interval $w_{c}$ is a value that considers the analog–digital converter (ADC) sampling rate $f_{s}$ and Δt, as well as the time taken to perform the frequency sweep. It is calculated as $w_{c}=∆tf_{s}$. Figure 3 shows the segmentation results in the time domain and spectrogram.

### 3.2. Code Classification Using Faster Region-Based CNN

Faster R-CNN [33,42] consists of a convolutional neural network (CNN) backbone to obtain the features of segmented spectrogram signals, a region proposal network (RPN) to propose a time–frequency domain region, and a classification network to classify the code and heterogeneous signal. The CNN backbone receives the segmented spectrogram of the received ultrasonic signal as the input and extracts the spatial features through convolution. When ultrasonic waves propagate through air, the power of the received signal is attenuated by distance and radiation. Therefore, the signal attenuation is corrected using a time gain amplifier, and the data are normalized to achieve a zero mean before input. The horizontal and vertical axes of the image are the time and frequency domains, respectively. The input image is selected with a size of 64 × 64, covering the time range ∆t and frequency range ∆f of a one-bit signal. The size of the input image is appropriately adjusted according to the sampling rate of the ADC, the window size N, and the overlap interval when converting to a spectrogram. The input volume was set to 64 × 64 × 3 because the signal power of the spectrogram is expressed in RGB colors. A pre-trained ResNet-50 is used as the CNN backbone. A residual neural network (ResNet) [43] is a CNN that solves the vanishing gradient problem using residual blocks. The ResNet architecture comes in various depths; ResNet-50 consists of 50 layers.

The RPN receives the features of the CNN backbone and proposes the time–frequency region of the received ultrasonic signal and the objectness score of the signal in that region. The RPN reduces the dimensionality of the input features and proposes regions through two layers for classification and bounding box regression, respectively. A two-dimensional window is stridden, and bounding boxes with three scales and three aspect ratios (1:1, 1:2, and 2:1) are used for every sliding position to find the bounding box in the features. For each bounding box, the regression layer outputs the position of the bounding box as the center x and y coordinates’ width and height, while the classification layer outputs the objectness score of the bounding box. The RPN performs region proposals for some bounding boxes with high objectness scores.

The classification network receives the features of the CNN backbone and the region proposal of the RPN and classifies the signal within the bounding box. Each bounding box is resized to a fixed-size feature vector using the region of interest (ROI) pooling layer, as the size of the features must be the same to perform the classification. For each ROI, the regression layer outputs better-fit bounding box information, and the classification layer outputs the code of the received signal and its probability or its probability of being a heterogeneous signal.

### 3.3. Training Dataset

The training dataset was acquired using the ultrasonic transmission and reception environment. The data were collected and classified into four cases. In the first case, only one bit is received without overlap. There are $2^{1}$ classes with code ‘0’ or code ‘1’. In the second case, two bits are received with overlap. There are $2^{2}$ cases of permutation with the repetition of codes ‘0’ and ‘1’. There are $2^{2}\times 5$ classes with an overlap ratio of 0.1, 0.3, 0.5, 0.7, and 0.9 for each case. Regarding the overlap, there are in total five classes, but the data were collected with a uniform random overlap ratio in the range of ±0.1 to increase the diversity of constructive–destructive interference according to the phase difference between the two signals. In the third case, three bits are received with overlap. There are $2^{3}$ cases of permutation with the repetition of code ‘0’ and code ‘1’. As in the second case, there are five overlap ratios and $2^{3}\times 5$ classes. The last case is one in which a heterogeneous signal is received. There are two classes: OOK signal and FSK signal. PSK has a similar spectrogram to OOK and was not classified separately.

The dataset has 100 elements for each class, and the signal power ratio and background noise were adjusted to prevent overfitting and increase the amount of data. The signal power ratio was adjusted by fine-tuning the distance of the two transmitters in five steps, from 0.2 to 1, in increments of 0.2. White Gaussian background noise was added to adjust the SNR in five steps, from 2 to 10 in two increments. Overall, the data have 2500 elements for each class.

### 3.4. Grouping and Merging

The classes vary depending on the overlap ratio or overlapping signals, but if segmentation is performed properly, only the code information of the bit located at the center needs to be obtained. Therefore, each class can be grouped into the code ‘1’ set $G_{1}$ or the code ‘0’ set $G_{0}$ according to the code information of the bit located at the center. In addition, certain classes are grouped into a set $G_{b}$ of both detected cases in which code ‘0’ and code ‘1’ signals are received simultaneously, and cases of heterogeneous signals and cases that are not detected are grouped into a non-detected set $G_{n}$. The signal $C_{rx,1}^{s}\left[n\right]$ demodulated to code ‘1’ and the signal $C_{rx,0}^{s}\left[n\right]$ demodulated to code ‘0’ for each group are as follows.

(10) $$C_{rx,1}^{s}\left[n\right]=\left\{\begin{array}{ll} 1 & ,\left\{G_{1},G_{b}\right\}⊃F\left(B_{n}^{s}\right)\\ 0 & ,Others \end{array}\right.\;C_{rx,0}^{s}\left[n\right]=\left\{\begin{array}{ll} 1 & ,\left\{G_{0},G_{b}\right\}⊃F\left(B_{n}^{s}\right)\\ 0 & ,Others \end{array}\right.$$

where the superscript s indicates whether the signals separated during the segmentation process are front or rear signals. Function $F\left(x\right)$ is a Faster R-CNN classifier that receives segmented spectrograms and outputs classified classes. If the n-th bit of the received signal is classified into a class included in the set $G_{1}$ corresponding to code ‘1’ and the both-detect case set $G_{b}$, the n-th bit of $C_{rx,1}$ is 1; otherwise, it is 0. If the n-th bit of the received signal is classified into a class included in the set $G_{0}$ corresponding to code ‘0’ and the both detected case set $G_{b}$, the n-th bit of $C_{rx,0}$ is 1; otherwise, it is 0. Therefore, for the both detected set $G_{b}$, $C_{rx,1}$ and $C_{rx,0}$ are both 1 in the corresponding bit, and for the non-detected set $G_{n}$, $C_{rx,1}$ and $C_{rx,0}$ are both 0 in the corresponding bit.

### 3.5. Code Division Multiple Access Decoding

The demodulated signal $C_{rx,1}$ indicates that code ‘1’ is received, and $C_{rx,0}$ indicates that code ‘0’ is received. Therefore, $C_{rx,1}$ is compared with the ID code $C_{ID}$, and $C_{rx,0}$ is compared with the inverted ID code, $1-C_{ID}$. Since the demodulated signal and the ID code have the same dimension, the similarity between the demodulated signal and the ID code can be obtained by performing the product sum. The similarity $\rho_{c1}$ for code ‘1’, the similarity $\rho_{c0}$ for code ‘0’, and the similarity $\rho_{c}$ between the ID code and the demodulated signal, which can be obtained from the sum of the two, are expressed in the equations below.

(11) $$\rho_{c1}=\sum_{n=1}^{L_{ID}}C_{ID}\left[n\right]C_{rx,1}\left[n\right]\;\rho_{c0}=\sum_{n=1}^{L_{ID}}\left(1-C_{ID}\left[n\right]\right)C_{rx,0}\left[n\right]\;\rho_{c}=\rho_{c1}+\rho_{c0}$$

If the similarity $\rho_{c}$ between the ID code and the demodulated signal is equal to the length $L_{c}$ of the ID code, the corresponding code has been detected, and the TOF is calculated. The TOF is calculated when the front signal $t_{f}$ and the rear signal $t_{r}$ are detected as follows, respectively.

(12) $$t_{f}=n_{s}/f_{s}$$

(13) $$t_{r}=\left(n_{e}-L_{c}w_{c}\right)/f_{s}$$

## 4. Experimental Results

### 4.1. Experimental Environment

**Figure 4:**
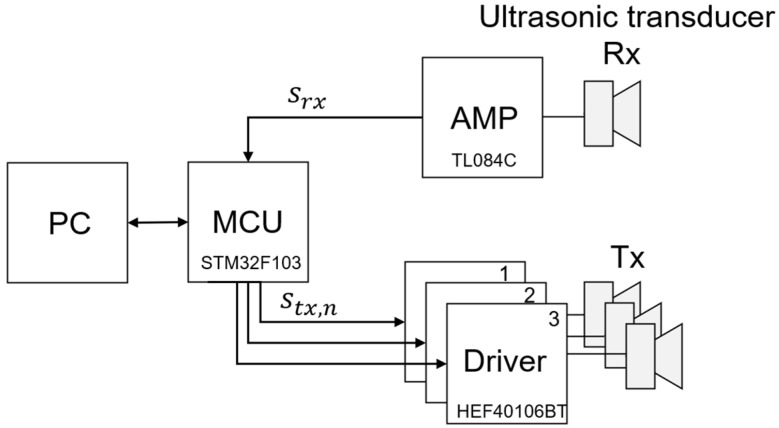
Ultrasonic transceiver system block diagram.

An ultrasound transmission and reception system was configured in a laboratory environment to verify the frequency sweep keying method using the Faster R-CNN proposed in this paper. As shown in Figure 4, an ultrasonic signal crosstalk environment was configured by connecting a single ultrasonic receiver and multiple ultrasonic transmitters to the micro controller unit (MCU). Ultrasonic transmitters have an individual time delay for their transmitted signals. Therefore, when combining signals, the constructive–destructive interference according to the phase and the overlap ratio of each transmission signal can be adjusted. The received signal data are collected through the amplifier and the ADC of the MCU. The collected data are transferred to a PC, and the demodulation and decoding processes are performed.

**Figure 5:**
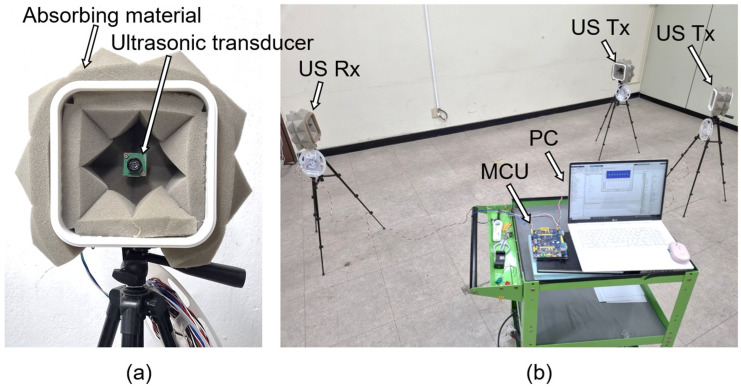
Ultrasonic transmission/reception environment: (**a**) ultrasonic transducer; (**b**) experimental environment.

Figure 5a shows the ultrasonic transmitting and receiving equipment used in the experiment. The used ultrasonic sensor was the Hagisonic HG-L40DC with a center frequency of 40 kHz, a bandwidth of 4 kHz, and a beam angle of 65°. In addition, ultrasonic-sound-absorbing material was attached around the ultrasonic sensor to minimize reverberation and multi-path fading other than the line of sight. As shown in Figure 5b, an ultrasonic signal crosstalk environment was configured, with one receiving device and multiple transmitting devices in an indoor environment measuring 10 m in width and 10 m in length. Table 1 lists the parameters used in the experiment.

### 4.2. Faster Region-Based CNN Classifier Validation

**Figure 6:**
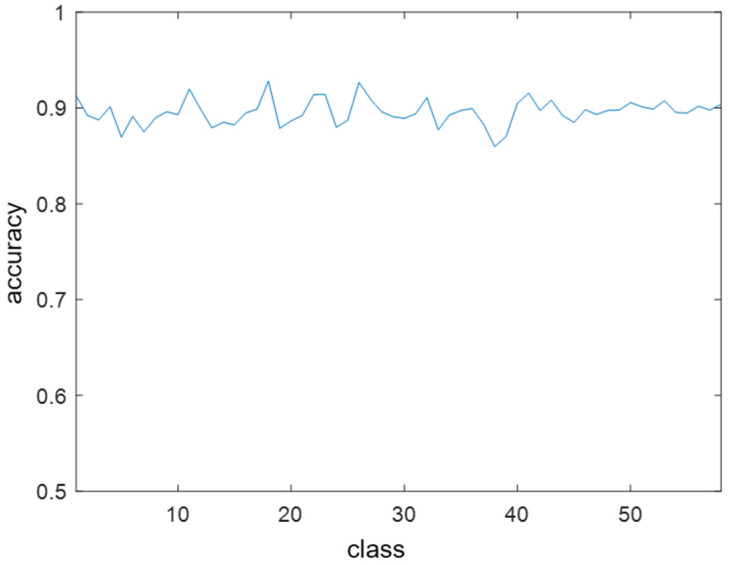
Precision of each class as a validation result.

The performance of the trained Faster R-CNN classifier was verified using five-fold cross-validation [44], which involved randomly dividing the data into five equal-sized subsets and using one subset as a validation set and the remaining four subsets as a training set. The process was repeated five times. Each of the five subsets was used once as a validation set. Figure 6 shows the precision of each class as a validation result. Precision is one of the classification performance indicators that is calculated based on the true positives (TP) and false positives (FP), as follows.

(14) $$PRECISION=\frac{TP}{TP+FP}$$

The verification results showed an average precision of approximately 0.9. As a result of analyzing the misclassification cases, most cases included classification errors because of the overlap ratio. The classification error in the overlap ratio did not affect the final demodulation performance because the bit information at the center was used in the subsequent grouping process. This showed that the Faster R-CNN classifier was properly trained.

### 4.3. Robustness Comparison for Homogeneous Signal with Other Modulation Methods

The performance of the frequency sweep keying method using the Faster R-CNN proposed in this paper was compared with the traditional modulation methods OOK, FSK, and PSK and the correlation-based frequency sweep keying method previously proposed by our research team. For comparison with other modulation methods, OOK used the modulation/demodulation method of [5], FSK used the modulation/demodulation method of [33], and PSK used the modulation/demodulation method of [34]. The root mean square (RMS) and standard deviation of the TOF error were measured in the millisecond scale. Ultrasound was transmitted from two transmitting nodes, and the signal was received by one receiving node. The position of the ultrasonic node was set so that the two transmitted signals were received with overlap. The manipulation variables of the experiment were the CDMA code length and overlap ratio. The CDMA code length was 2, 4, 8, 16, 32, 64, and 128; the overlap ratio was 0 to 1 in 0.1 increments, and each case was tested 100 times. The experimental results are expressed in two forms: TOF error according to the code length and detection rates according to the overlap ratio. Table 2 lists the TOF error results of each modulation method according to the code length. FSWK-corr represents the previous correlation-based frequency sweep keying method. The code detection rate was low for OOK, FSK, and PSK, with a short code length of eight or less, resulting in a substantial TOF error. Meanwhile, for PSK, even when the code length was long, the code detection rate was low and the TOF error was large. When the frequency sweep keying method was used, the TOF error was relatively small due to the high code detection rate, even if the code length was short. Comparing the proposed method with FSWK-corr, the proposed method estimated the TOF in the time domain, so the TOF error was small. However, the TOF errors of the proposed method and OOK were similar when the code length was long.

The performance of the demodulator and the CDMA decoder was compared and evaluated against other methods for the purpose of verification. If errors occur during the demodulation process, the orthogonality factor deteriorates and the errors propagate to the detection error rate, which is the result of CDMA decoding. Therefore, in this paper, the demodulated code error rate (DCER) is defined as a quantitative measure to evaluate demodulator performance compared to other methods, as follows.

(15) $$DCER=\frac{\sum C_{ID}⊕C_{rx}}{L_{ID}}$$

DCER is a normalized value obtained by dividing the total sum of exclusive disjunctions of ID code $C_{ID}$ and demodulated code $C_{rx}$ by $L_{ID}$. DCER has a value of zero when no demodulation error occurs and a value of one when errors occur in all bits.

**Figure 7:**
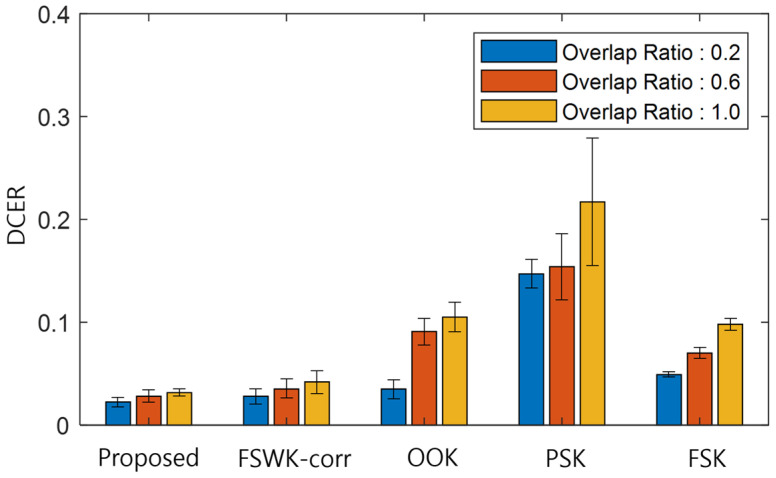
Results of comparing the DCER according to the overlap ratio.

Figure 7 presents an evaluation of the DCER of each method according to the overlap ratio of 0.2, 0.6, and 1.0. The error bar represents the standard deviation of the error rate. The proposed method had a small error rate of less than 0.05, regardless of the overlap ratio. It also led to error rates approximately 25% lower than that of the same modulation method, FSWK-corr, and had a smaller standard deviation range, making it more stable. In the case of OOK and FSK, the error rates reached approximately 0.1, and FSK had a smaller standard deviation range. In the case of PSK, errors in phase information were caused by the overlap of signals with different strengths, and the demodulation error rates were generally high.

**Figure 8:**
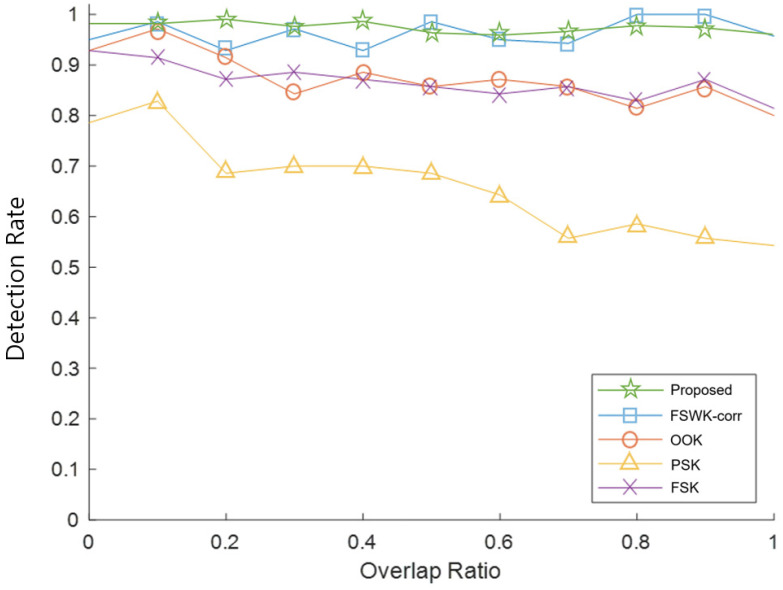
Results of comparing the detection rates according to the overlap ratio.

Figure 8 shows the results of comparing the detection rates according to the overlap ratio. Methods using frequency sweep keying, which include the proposed method and FSWK-corr, showed detection rates of more than 0.93, regardless of the overlap ratio. For OOK and FSK, however, as the overlap ratio increased, the detection rates decreased from approximately 0.92 to approximately 0.84, and the detection rates were even lower for PSK. Therefore, the proposed method proved robust to overlap and had a small TOF error compared to other methods.

### 4.4. Robustness Comparison for Heterogeneous Signal

Table 3 compares the robustness of the demodulator to heterogeneous signals. Each keying method exhibited different characteristics associated with the demodulation of heterogeneous signals. The OOK demodulator usually estimated code ‘1’ for all heterogeneous signals. The PSK demodulator estimated the OOK signal as code ‘1’ because their phases were identical, leading to false detections. The PSK demodulator estimated the FSK and frequency sweep keying signals as random codes because of their continuously changing phases, also leading to false detections. The FSK demodulator failed to detect the OOK and PSK signals because they only had center frequency components and caused false detection for the frequency sweep keying signals. The correlation-based frequency sweep keying demodulator falsely detected codes ‘0’ and ‘1’ for the OOK signal with code ‘1’ and the PSK signal, while it failed to detect the OOK signal with code ‘0’. The correlation-based frequency sweep keying demodulator falsely detected code ‘1’ or code ‘0’ for the FSK signal because the frequency rose or fell when the code changed. For the demodulator using Faster R-CNN, which is the proposed method, the OOK and PSK signals used only the center frequency, so they are recognized as horizontal parallel bars in the spectrogram and were classified as heterogeneous signals. False detection occurred in approximately 1% of the OOK and PSK signals. For the FSK signal, when the position at which the code changes was segmented, the frequency rose or fell, resulting in an approximately 21% false detection rate as code ‘0’ or ‘1’.

## 5. Conclusions

As the number of devices using ultrasonic sensors increases, the crosstalk between ultrasonic nodes becomes more frequent. Crosstalk includes situations in which signals overlap and heterogeneous signals are received. To solve this problem, our research team proposed a frequency sweep keying method based on correlation in the time–frequency spectrogram in a previous study. The suggested correlation-based method was more robust to overlap than the existing method. However, it had a high false detection rate when heterogeneous signals were received. In addition, the time resolution was lower than that of other methods because it performs CDMA on the spectrogram. This study proposed a method robust to overlap using the frequency sweep keying method and demodulated the signal using Faster R-CNN to increase the detection rate of proper signals by ignoring when heterogeneous signals are received. An experiment implementing an environment in which ultrasonic crosstalk occurs showed that the detection rate was higher than other traditional modulation methods at a short code length, and the TOF error was reduced by approximately 75% compared to the correlation-based frequency sweep keying method. It showed a high non-detection rate for heterogeneous signal input and exhibited good performance in demodulating only homogeneous signals. Based on the experimental results, the proposed method was robust against overlap and successfully performed CDMA even with a short code length. Therefore, it has a short dead zone and is robust to heterogeneous signals, highlighting its potential in ultrasonic sensors for blind spot detection in automobiles.

On the other hand, several problems have yet to be overcome before this method can be applied successfully. In this study, experiments were conducted in an ideal environment to verify the method’s performance in crosstalk situations. Nevertheless, the proposed method may have limitations when well-known problems of ultrasonic sensing, such as multi-path interference, reverberation artifacts, and reflected signal distortion due to object shape, occur. The performance of the proposed method is greatly affected by the training dataset of Faster R-CNN. If the above problem occurs, the shape of the spectrogram of the received signal may change and misclassification may become more likely. To solve this problem without significantly changing the proposed method, it is possible to train Faster R-CNN more robustly by training on received data in various situations in the training dataset.

## Figures and Tables

**Table 1:** Experimental parameters.

Parameter	Description	Value	Unit
$f_{c}$	Ultrasonic transducer center frequency	40	kHz
	Ultrasonic transducer frequency bandwidth	4	kHz
$f_{s}$	ADC sampling rate	1	MHz
∆f	Sweep frequency range	8	kHz
∆t	Sweep time	2	ms

**Table 2:** Comparison of the TOF error according to the ID length of the proposed method and the existing methods. Measured in millisecond scale.

Code Length	Proposed	FSWK-Corr	OOK	FSK	PSK
2	0.061 ± 0.019	0.362 ± 0.073	66.792 ± 29.135	66.268 ± 29.847	68.441 ± 30.362
4	0.064 ± 0.020	0.324 ± 0.059	32.235 ± 15.349	32.379 ± 15.894	33.618 ± 16.168
8	0.062 ± 0.019	0.326 ± 0.121	3.215 ± 1.548	4.782 ± 2.161	11.272 ± 4.558
16	0.064 ± 0.019	0.272 ± 0.075	2.371 ± 1.299	4.185 ± 2.335	12.185 ± 5.654
32	0.059 ± 0.018	0.249 ± 0.043	0.548 ± 0.024	1.335 ± 0.887	19.143 ± 8.548
64	0.058 ± 0.017	0.314 ± 0.054	0.064 ± 0.019	0.248 ± 0.052	21.151 ± 10.167
128	0.057 ± 0.017	0.291 ± 0.039	0.059 ± 0.018	0.206 ± 0.057	16.688 ± 7.748

**Table 3:** Results of comparing the robustness of demodulator to heterogeneous signals.

Modulation Method	Detection Rate/Wrong Detection Rate/Non-Detection Rate				
	Faster R-CNN	FSWK-corr	OOK	PSK	FSK
Proposed	0.99/0.01/0.00	0.97/0.00/0.03	0.43/0.57/0.00	0.69/0.31/0.00	0.61/0.39/0.00
OOK	0.01/0.01/0.99	0.26/0.30/0.44	0.94/0.06/0.00	0.53/0.47/0.00	0.44/0.56/0.00
PSK	0.00/0.01/0.98	0.46/0.54/0.00	0.51/0.49/0.00	0.96/0.04/0.00	0.52/0.48/0.00
FSK	0.14/0.07/0.79	0.31/0.42/0.27	0.47/0.53/0.00	0.42/0.58/0.00	0.97/0.03/0.00

## Data Availability

Data are contained within the article.
